# HRM and CRAC in MxIRT1 act as iron sensors to determine MxIRT1 vesicle-PM fusion and metal transport

**DOI:** 10.1080/15592324.2021.2005881

**Published:** 2021-11-23

**Authors:** Song Tan, Xi Zhang, Qi Zhang, Yu-Meng Li, Peng Zhang, Li-Ping Yin

**Affiliations:** a School of Pharmacy, Anhui University of Chinese Medicine, Hefei, China; b College of Life Science, Capital Normal University, Beijing, China

**Keywords:** Histidine rich motif, CRAC motif, iron, iron sensor, MxIRT1, detergent-resistant membrane

## Abstract

The IRON-REGULATED TRANSPORTER1 (IRT1) is critical for iron uptake in roots, and its exocytosis to the plasma membrane (PM) is regulated by detergent-resistant membranes. However, studies on IRT1 exocytosis and function in response to iron status are limited. Presently, we found that the histidine-rich motif (HRM) of MxIRT1 could bind to iron directly and HRM determined the delivery of MxIRT1 to the PM, after which the cholesterol recognition amino acid consensus (CRAC) motif-regulated MxIRT1 mediated metal transport. IMAC assay revealed that H192 was the vital site for HRM binding to Fe^2+^, and metal-binding activity was stopped after the deletion of HRM (MxIRT1∆HM) or in H192 site-directed mutants (H_192_A). MxIRT1∆HM or H_192_A in transgenic yeast and Arabidopsis failed to localize in the PM and displayed impaired iron absorption. In the PM, Y266 in CRAC was required for metal transport; Y266A transgenic Arabidopsis displayed the same root length, Cd^2+^ flux, and Fe concentration as Arabidopsis mutant *irt1* under iron-deficient conditions. Therefore, H192 in HRM may be an iron sensor to regulate delivery of MxIRT1 vesicles to the PM after binding with iron; Y266 in CRAC acts as an iron sensor for active metal transport under iron-deficient conditions.

## Introduction

Iron is a key transition element in the biosphere, and it is crucial for living organisms because it participates as a redox cofactor in a variety of vital processes, including respiration and photosynthesis.^1^ Iron bioavailability in plants is often limited, as it is present in the form of insoluble complexes in calcareous soils. Therefore, iron is a limiting factor for plant biomass production and hence is required as an important component for agricultural productivity. Transporters are at the center of regulatory modules, allowing optimal assimilation, distribution, or efflux of substrate molecules. IRON-REGULATED TRANSPORTER1 (IRT1) is the major player in the regulation of plant iron homeostasis, as attested by severe chlorosis and the lethality of the *irt1-1* knockout mutant.^2^ The precise control of transport proteins requires sophisticated sensing and signaling mechanisms to perceive specific cues, such as substrate concentration or environmental conditions (e.g., salt, drought, wounding, etc.), and the transduction of this information regulates transporter gene expression. After decades of research on IRT1, detailed regulatory networks have been shown to integrate several endogenous and exogenous cues at various levels to sense iron conditions and regulate IRT1 expression, thereby fine-tuning iron uptake.^3,4^

At the transcriptional level, the expression of *IRT1*, along with that of *FRO2* and *AHA2* genes in Arabidopsis, is activated under iron-limited conditions through the direct action of the basic helix-loop-helix (bHLH) transcription factor FER-like iron deficiency-induced transcription factor (FIT), which can form heterodimers with other bHLH proteins.^5^ In the plasma membrane (PM), the C2 domain-containing peripheral membrane protein ENHANCED BENDING1 (EHB1) could directly interact with the cytoplasmically exposed variable region of IRT1, an interaction promoted by the presence of calcium. EHB1 could also act as a direct inhibitor of IRT1-mediated iron import into the cell.^6^ Recent studies have also shown that IRT1 is not only the final target of these regulatory networks but also plays a key role in metal sensing.^7^ Rather, *IRT1* is post-translationally regulated by divalent, non-iron metals (Zn, Mn, Co), which are also substrates for IRT1 due to their similarity with iron. These metals also massively accumulate in plant cells upon iron deficiency.^2,8^ IRT1 is gradually removed from the PM through a monoubiquitin- and clathrin-dependent mechanism via differential ubiquitination on exposure to increasing concentrations of these highly reactive non-iron metals. Moderate non-iron metal concentrations trigger multimonoubiquitination of IRT1 on lysine (K) at K154 and K179 via an unknown PM localized E3 Ub ligase.^7,9,10^ This triggers IRT1 internalization toward early endosomes and decreases the pool of the transporter available in the PM. Higher concentrations of non-iron metals lead to the extension of multimonoubiquitination into K63-linked polyUb chains via the RING E3 Ub ligase IRON DEGRADATION FACTOR1 (IDF1),^11^ and presumably by E2 UB-CONJUGATING ENZYMES35/36 (UBC35/36), which are responsible for K63 polyUb chain formation in Arabidopsis.^12^ The phosphorylation of the target to be ubiquitinated often triggers the recruitment of E3 Ub ligases. IRT1 follows this rule via the recruitment of IDF1 to multimonoubiquitinated IRT1 upon detection of metal excess, which requires phosphorylation of IRT1 at serine/threonine residues neighboring Ub sites. This is achieved via the CIPK23 kinase, a well-known kinase that phosphorylates other plant transport proteins such as HIGH AFFINITY K^+^ TRANSPORTER1.1 (HAK5), CHLORATE RESISTANT1/NITRATE TRANSPORTER1.1 (CHL1/NRT1.1), and AMMONIUM TRANSPORTER1 (AMT1).^13–15^ K63 polyubiquitination triggers IRT1 migration toward late endosomes and likely alters the balance between vacuolar targeting and recycling to push IRT1 toward the lytic vacuole for degradation. In late endosomes, the late endosomal protein SORTING NEXIN1 (SNX1) can control the fate of IRT1 by promoting its recycling to the cell surface.^16^ FYVE1, a phosphatidylinositol-3-phosphate-binding protein recruited to late endosomes, controls IRT1 recycling to the plasma membrane and impacts the polar delivery of this transporter to the outer plasma membrane domain.^10^

In our previous study, we cloned a high-efficiency Fe^2+^ transporter, MxIRT1, from *Malus xiaojinensis* roots,^17^ that fuses with the PM in response to iron supplementation,^18^ and is then degraded through autophagy in the presence of excess iron.^19^ Fusion and autophagy are key regulatory steps for high-affinity iron transport that allow plants to sense and respond to iron conditions. However, many IRT1 regulatory mechanisms remain unknown; for example, it is unclear how vesicles containing IRT1 are recruited to the PM or which proteins sense and respond to metal concentration changes in the environment to initiate exocytosis and membrane fusion. Further research is necessary to elucidate this regulation. MxIRT1 has been identified as a ZIP family protein that contains all the conserved domains of this family. It has been suggested that the His rich motif (HRM) found in many ZIP family members might serve as an intracellular metal-binding/sensing domain that could mediate posttranslational regulation.^7,8^ When His residues in MxIRT1 were replaced by Ala, some of the resultant mutants, such as H_2_A and H_3,4_A, could not rescue the iron uptake mutant DEY1453 (*fet3fet4*), indicating that these His residues are essential for iron transport, while H_1_A exhibits no differences from WT in function.^20^ These results suggest that the His-rich domain in MxIRT1, even in some of these sites, in the His-box might be involved in transporting iron. However, the direct binding result of the loop with iron has still not been identified, and how MxIRT1 senses the iron condition to regulate the fusion of MxIRT1 vesicles with PM remains unknown.

In this study, we found that the HRM in MxIRT1, specifically H192, was necessary for MxIRT1 binding to iron and targeting to the PM. Deletion of the HRM of MxIRT1 (MxIRT1∆HM) resulted in loss of ability to rescue the growth and metal transport of Arabidopsis mutant *irt1*. Site-directed mutations in HRM, including H_184_A, H_192_A, and H_184-192_A, revealed that H_192_ was the key site regulating the fusion of MxIRT1 with the PM based on functional analysis and localization in yeast and plants. Thus, the HRM acted as an iron sensor to sense environmental iron and regulate MxIRT1 vesicle-PM fusion, and H192 specifically was the critical determinant. We further confirmed that the CRAC motif Y266 is necessary to activate metal transport in transgenic Arabidopsis after moving from detergent-resistant membranes (DRMs) to non-DRMs in the PM under iron-deficient conditions. Taken together, these results show that the HRM affects the fusion of MxIRT1 with the PM by binding with iron directly to sense iron changes, after which CRAC regulates metal transport functions in the PM.

## Materials and Methods

### Yeast strains, transformation and growth conditions

Yeast mutants DEY1453 were generous gifts from Professor Nicolaus von Wiren (Department of Physiology and Cell Biology, IPK Gatersleben, Germany). Wild-type cell was grown in YPD medium (2 g/L peptone, 1 g/L yeast extract and 2 g/L glucose). Transgenic yeast cell was grown in SD-Ura− medium consisting of SD (6.7 g/L yeast nitrogen base, 0.77 g/L lacking uracil DO supplement) and 0.2 g/L glucose.

The histidine-rich motif deletion mutant was conducted in three step: In first step, getting fragment I by PCR using primers MxIRT1-F and H (Up-down), and template pYES2.0-MxIRT1-GFP; Getting fragment II using primers MxIRT1-R and H (Down-up), and template pYES2.0-MxIRT1-GFP. In second step, mixing fragment I and fragment II, and PCR using primers MxIRT1-F and MxIRT1-R. In third step, getting the largest fragment after DNA gel recycle, and inserting into pYES-GFP. The histidine-rich motif site-directed mutants were conducted by PCR using the PrimeSTAR Max DNA Polymerase and primer pairs Hm-F and Hm-R, H_184_A-F and H_184_A-R, H_192_A-F and H_192_A-R, H_184/192_A-F and H_184/192_A-R, H_184-192_A-F and H_184-192_A-R using pYES2.0-MxIRT1-GFP as template. The CRAC motif site-directed mutants were conducted by PCR using the PrimeSTAR Max DNA Polymerase and primer pairs L262A-F and L262A-R, Y266A-F and Y266A-R, K270A-F and K270A-R using pCambia1302-MxIRT1-GFP as template. The primer sequences are listed in Table S1. The plasmid was transformed into yeast cell using the highly efficient Li-acetate transformation method and the transformed yeast cell was grown on SD-Ura− medium.

In the complementation and protein detection assays, glucose was replaced by galactose to drive the protein expression by the galactose promoter (GAL1) present in the pYES2.0 vector. Yeast cell was diluted to optical densities of 0.1, 0.01, 0.001 and 0.0001. Thereafter, 4 μL of each cell suspension was dropped on SD medium lacking uracil and supplemented with 30 μmol L^−1^ Fe–Na–EDTA for Fe-sufficient treatments or 30 μmol L^−1^ BPDS (batho-phenanthroline disulfonic acid) for the Fe-limited treatments. The plates were incubated at 28℃ for 3 days.

Fe concentration in yeast cell was measured according to the previously described method.^21^ The induced yeast cell was cleaned, divided into three groups and dried before being weighed, it was then treated with nitric acid and measured by inductively coupled plasma mass spectrometry (ICP-MS).

### Rice protoplasts and Arabidopsis preparation, transformation and growth conditions

The *irt1* Arabidopsis mutant was a gift from Professor Yeh Kuo-Chen (Agricultural Biotechnology Research Center, Academia Sinica, Taipei, Taiwan). The plant transformation was performed as described previously.^11,22,23^ Wild type and *irt* mutant seeds were sterilized, placed in the dark at 4°C for 2 days, and then sown on 1/2 MS medium plates supplemented with 2% sucrose and 1% agar, pH 5.8. Transgenic plants were grown on plates supplemented with hygromycin (20 g/mL). Plates were incubated at 23°C for 3 days, seedlings were transferred to the normal 1/2 MS medium plates or the iron deficient 1/2 MS medium plates for 7 days or soil to compare phenotype. Rice protoplast isolation and transformation is shown in previous reports.^24^

### Observation of subcellular localization of protein in yeast, rice protoplasts and Arabidopsis seedlings

Confocal microscopy was performed on an LSM 780 (Zeiss). For green fluorescent protein (GFP) visualization, excitation at 488 nm and detection between 505 and 545 nm were used. For FM4-64, excitation at 561 nm and detection from 575 to 615 nm were used. Pinholes for both channels were set to 1 Airy Unit resulting in optical slices of 0.8 mm. Images were recorded in a 1024 pixel format. Colocalization analysis was performed on 8-bit gray-scale image pairs, representing the GFP and FM4-64 channels. Images were loaded in ImageJ software (http://rsb.info.nih.gov/ij) and analyzed using the JACoP v2.0 plug-in. Threshold values were automatically adjusted by the software.

### Metal-binding analysis using immobilized metal ion affinity chromatography (IMAC)

The MxIRT1 derivative-GST fusion plasmid, as well as GST-vector as a negative control and IDEF1-GST fusion plasmid as a positive control, was introduced into E. coli strain BL21 (DE3) pLysS. Recombinant protein was obtained and purified using the GST fusion system according to previous report.^25^ The metal-binding capacity of the MxIRT1 derivatives is analyzed as follows. Bio-Spin empty columns (Bio-Rad, http://www. bio-rad.com/) were first embedded with about 100 μL of iminodiacetic acid (IDA) agarose resin, and subsequently charged with 500 μl of 50 mM FeSO_4_ with 150 mM sodium ascorbate (Fe^2+^). After washing with binding buffer (5 mM imidazole, 500 mM NaCl, 20 mM Tris–HCl, pH 7.9), 800–1120 μl of protein solution containing about 25–100 μg of MxIRT1 derivatives in binding buffer was applied to the columns, and the flow through fraction was collected. Unbound and bound protein was serially washed out and collected in each fraction by applying 300 μl each of binding buffer containing 5, 30, 60, 250, or 1000 mM of imidazole. For Fe^2+^ assay, each binding buffer solution was supplemented with 25 mM sodium ascorbate (final concentration) to prevent oxidation of Fe^2+^. Aliquots (20 μl of input, 53 μl of flow through, and 80 μl each of the other fractions) were subjected to SDS-PAGE and detected using Flamingo Fluorescent Gel Stain (Bio-Rad) and Typhoon 9400 detector.

### Leaf chlorophyll content measured in transgenic Arbidopsis, and Cd^2+^ flux measured by noninvasive microtest technique (NMT) in Arabidopsis root

A SPAD-502 chlorophyll meter was utilized to measure chlorophyll content.^26^ The net Cd^2+^ flux in Arabidopsis root was measured using a noninvasive Micro-test Technology (NMT®; Xuyue Science and Technology Co., Ltd.) as previously reported.^27^ The transformed Arabidopsis seedlings were measured in the testing solution, which contained 0.05 mM CdCl_2_. The microelectrode was then front filled with a column of a selective liquid ion-exchanger cocktail (LIX) imparting ion-selectivity with the column length of Cd^2+^ at 15–20 μm (St. Louis MO, USA). Cd^2+^ ion flux was calculated by Fick’s law of diffusion: J = -D (dc/dr), where J represents the ion flux (10^−12^ mol cm^−2^ s^−1^), dc is the ion concentration difference (10^−3^ mol/L), dr is the distance between two measured points (μm), and D is the ion diffusion constant in a particular solution and tem perature (cm^2^/s). Probing, data recording, digital image acquisition and calibrations were performed using the ASET software. All the data were statistically analyzed.

### Statistical analysis

All experiments were performed at least in triplicate. One-way ANOVA was used to compare the quantitative difference between the different samples. Values of *P* < .05 were considered statistically significant.

## Results

### Sequence alignment of His rich motif among the loop in IRT1s

**Figure 1.**
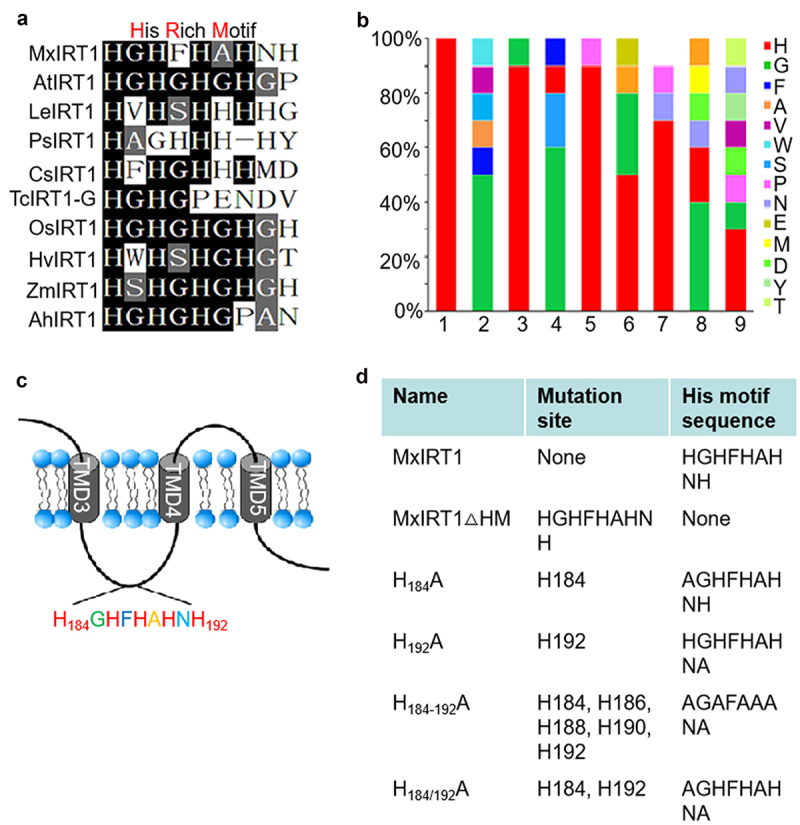
Sequence alignment of His rich motif (HRM) among the loop in IRT1s and schematic representation of MxIRT1 mutants.

The large hydrophilic cytosolic loop between transmembrane domain III (TMD3) and transmembrane domain IV (TMD4) of IRT1s is mainly oriented to the cytoplasm and has multiple functions.^4^ FYVE1, a phosphatidylinositol-3-phosphate-binding protein, interacts with the large loop, controls IRT1 recycling to the PM, and impacts the polar delivery of this transporter to the outer plasma membrane domain.^10^ IRT1 DEGRADATION FACTOR1 (IDF1) interacts with a large loop and is involved in IRT1 degradation.^11^ Among the large loops, histidine-rich motifs (HRM) are important, and site-directed mutagenesis of H_186_A or H_188/190_A in these motifs impairs metal transport ability.^20^ However, the detailed mechanism and process regulating HRM sensing of iron and how this affects IRT1 intracellular traffic or fusion with the PM remains unknown. The sequence alignment of HRMs among the loop in IRTs is highly conserved, in particular the first, second, and third histidine residues (Figure 1a). The first histidine, H184, is the most conserved site in HRM. The fifth histidine, H192, displayed the highest variation, and was only observed in MxIRT1 and OsIRT1 (Figure 1b). Mutation of MxIRT1 histidine residues H_186_A or H_188/190_A could reduce the ability of MxIRT1 to transport metal, but the function of the most variable residue, H_192_ has not been studied.^20^ To further analyze and confirm the function of HRM, we constructed an HRM deletion mutant of MxIRT1, MxIRT1∆HM, and the site-directed mutants H_184_A, H_192_A, H_184-192_A, and H_184/192_A, as shown in Figure 1c, d.

A, Sequence comparison of HRM between MxIRT1 and other IRT1s. Identical residues are shown on a black background, and analogous residues are on a gray background. No amino acid residues are replaced with “–”.B, The occurrence probability of amino acid residues among HRM in IRT1. The probability is quantified, and amino acids are represented by different colors. Values in the X axis represent the position of the amino acid in the HRM.C, The schematic representation of histidine site-directed mutants and HRM deletion Mutation (ΔHM) in MxIRT1. The residues to be mutated are marked by red on the left side and detailed sequence information is described in the right side.D, The name and sequence of HRM point and deletion mutants of MxIRT1.

### HRM affects iron transport of MxIRT1 by regulating the fusion of MxIRT1 vesicles with the PM in Arabidopsis thaliana

**Figure 2.**
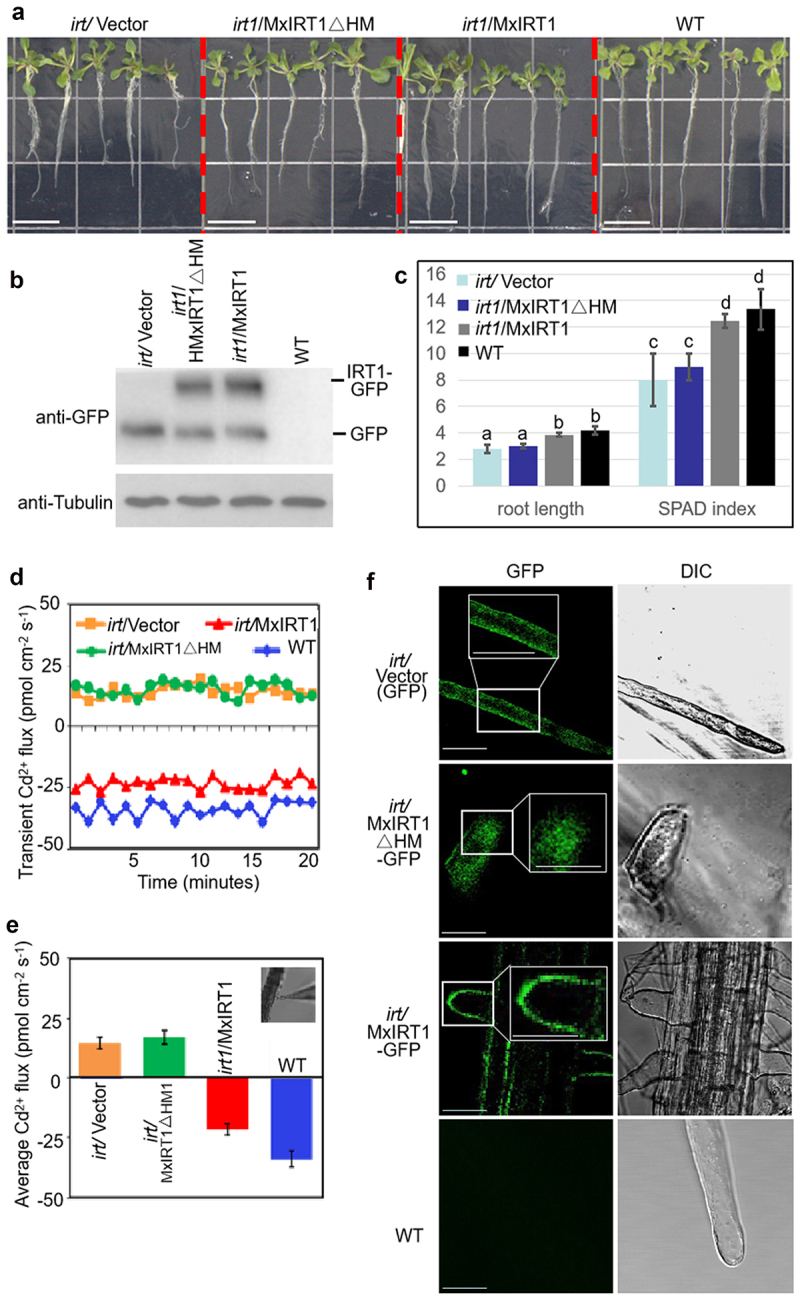
Phenotypic and functional comparison of Vector (GFP), MxIRT1ΔHM (MxIRT1ΔHM-GFP), MxIRT1 (MxIRT1-GFP) transgenic and WT (wild type) *Arabidopsis thaliana.*

To address the function of HRM, we constructed a Vector (GFP), and created MxIRT1∆HM (MxIRT1∆HM-GFP) and MxIRT1 (MxIRT1-GFP) transgenic Arabidopsis. As Arabidopsis mutant *irt1* displays retarded growth compared to WT, as previously reported,^2^ overexpressing MxIRT1 could rescue the growth of *irt*, but not Vector and MxIRT1∆HM (Figure 2a). Western blotting was used to detect the protein expression of the vector, MxIRT1∆HM and MxIRT1 in Arabidopsis using a GFP antibody (Figure 2b). MxIRT1 rescued the root length and SPAD index of *irt* mutants, but not Vector and MxIRT1∆HM (Figure 2c). The noninvasive microtest technique (NMT) has been extensively used to test ion flux in plants,^21,27^ and the Cd ion transient velocity was tested using NMT to compare Cd uptake rates among the transgenic Arabidopsis. The transient (Figure 2d) and average (Figure 2e) Cd^2+^ flux showed that WT and MxIRT1 could absorb of Cd^2+^, as the transient Cd flux in the root surface was negative. However, Vector and MxIRT1∆HM displayed Cd^2+^ efflux because the transient Cd flux in the root surface was positive. Confocal results in root hair showed that MxIRT1 was localized to the PM, while MxIRT1ΔHM lost the ability to target the PM (Figure 2f). This indicates that metal transport was impaired after HRM deletion. Thus, HRM is necessary for normal growth of MxIRT1, chlorophyll accumulation, and metal absorption, and is critical for MxIRT1 vesicle-PM fusion.

A, The phenotype of Vector, MxIRT1ΔHM, MxIRT1 transgenic and WT 7-day-old *Arabidopsis thaliana*. Bars = 1 cm.B, Western blotting (WB) detecting the expression of Vector, MxIRT1ΔHM, MxIRT1 using GFP antibody in transgenic *Arabidopsis thaliana*.C, The root length (cm) and chlorophyll content (SPAD index) of 7-day-old transgenic and WT *Arabidopsis thaliana*. The Soil and Plant Analyzer Development (SPAD) index of the chlorophyll content in *Arabidopsis thaliana* leaves was measured by SPAD-502 Chlorophyll Meter. Every independent measurement contains three replicates. Different letters indicate significant differences (*P* < .05) as determined by ANOVA.D, Presentation of transient Cd^2+^ flux by NMT in the transgenic and WT *Arabidopsis thaliana* roots in 0.05 mM CdCl_2_.E, Average Cd^2+^ flux by NMT in the transgenic and WT *Arabidopsis thaliana* roots. The image in the right corner shows the measuring position for root hair and Cd^2+^-selective microelectrode used in NMT. Every independent measurement contains >80 replicates. Bars represent mean ± standard error.F, Subcellular localization of Vector (GFP), MxIRT1ΔHM-GFP, MxIRT1-GFP in transgenic and WT *Arabidopsis thaliana*. GFP, green fluorescence protein indicated the location of fusion proteins; DIC, differential interference contrast. Bars = 10 µm.

### H192 in HRM is the critical site regulating the metal transport function of MxIRT1 and fusion with the PM in both WT and transgenic plants

**Figure 3.**
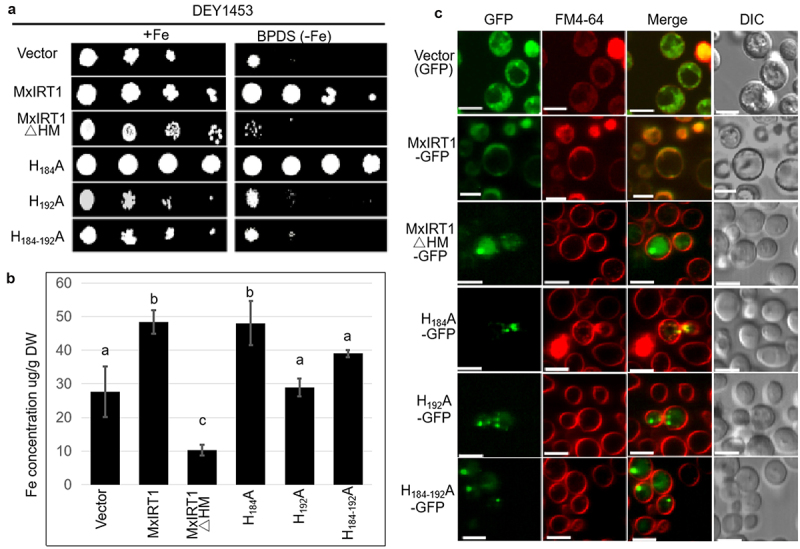
Functional analysis and PM localization of MxIRT1 HRM point mutants in yeast.

To further analyze the key site in HRM determining PM fusion, we constructed the site-directed mutants H_184_A, H_192_A, and H_184-192_A, and overexpressed these proteins both in yeast and transgenic Arabidopsis. Complementary functional analysis of MxIRT1∆HM in yeast revealed retarded growth under -Fe (30 μM BPDS) conditions (Figure 3a). H_184_A, which was the most conserved site in IRT1s, rescued the growth of yeast *fet3fet4* mutant DEY1453 under 30 μM BPDS conditions, similar to MxIRT1 (Figure 3a). The fifth histidine, H192, displayed the highest variation and was only observed in MxIRT1 and OsIRT1 (Figure 1b). H_192_A and H_184-192_A lost the ability to rescue the growth of DEY1453; the retarded growth was more remarkable in iron-deficient conditions, similar to MxIRT1∆HM. ICP-MS was used to measure Fe concentration in transgenic yeast. The iron concentration was low in MxIRT1∆HM, H_192_A, and H_184-192_A as compared to in MxIRT1 and H_184_A (Figure 3b). Thus, the metal transport function was impaired after H_192_A was replaced in transgenic yeast.

A, Yeast was diluted to an optical density of OD_600_ equal to 0.1, 0.01, 0.001 and 0.0001 and dotted (4 µL) on synthetic defined medium without uracil (SD-Ura medium) containing galactose at 28℃ for 3 days. Yeast mutant strain DEY1453 (*fet3fet4*) containing pYES2.0 (vector), MxIRT1, MxIRT1ΔHM and three MxIRT1 His site-directed mutagenesis vectors were grown on SD-Ura medium containing FeSO_4_ or ferrous chelates (BPDS).B, Iron content in transgenic yeast was measured by using inductively coupled plasma mass spectrometry (ICP-MS). Every independent measurement contains three replicates. Bars represent mean ± standard errors (n = 3). Different letters indicate significant differences (*P* < .05) as determined by ANOVA.C, Subcellular localization of Vector, MxIRT1, MxIRT1ΔHM and three MxIRT1 His site-directed mutants in yeast. GFP, green fluorescence protein indicates the location of fusion proteins; FM4-64, cell endomembrane system dye indicates of the cell membrane; Merge, image overlap showing both green fluorescence and red fluorescence; DIC, differential interference contrast. Fluorescence was observed after adding FM4-64 with 30 min. Scale bar, 5 µm.

Confocal results showed that MxIRT1 and H_184_A could target the PM in yeast (Figure 3c). Similar to MxIRT1∆HM, H_192_A and H_184-192_A lost the ability to target the PM. Thus, H192 in the loop is the vital site for regulating MxIRT1 vesicle fusion with the PM. These results indicate that H192 in HRM is required for the metal transport function of MxIRT1 and its fusion with the PM in yeast.

**Figure 4.**
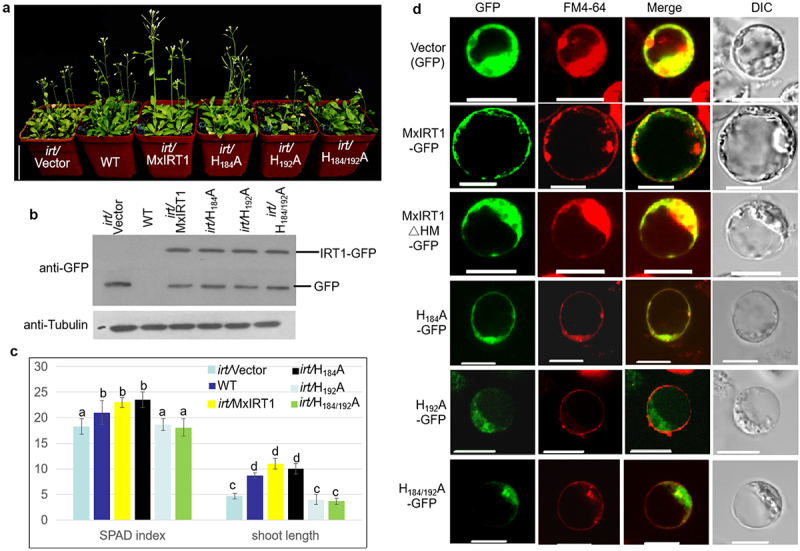
Functional analysis and PM localization of MxIRT1 HRM point mutants in plant.

The physiological role of the MxIRT1 point-site mutant in Arabidopsis was further investigated using transgenic Arabidopsis lines expressing the mutant version of the MxIRT1 gene. We overexpressed the site-directed mutants H_184_A, H_192_A, and H_184/192_A in Arabidopsis. Similar to transgenic yeast, H_184_A in transgenic Arabidopsis could rescue the growth of Arabidopsis mutant *irt1* under soil conditions (Figure 4a, FigureS1A), but H_192_A and H_184/192_A lost the ability to rescue the growth of *irt1*. Western blotting detected the protein expression of the Vectors, H_184_A, H_192_A, H_184/192_A, and MxIRT1 in Arabidopsis when using GFP antibody to confirm that these plants were transgenic (Figure 4b). The leaf chlorophyll accumulation (SPAD index) and shoot length of H_192_A and H_184/192_A were lower than those of H_184_A and MxIRT1 (Figure 4c). The root length (FigureS1B) and leaf area of H_192_A and H_184/192_A were lower than those of H_184_A and MxIRT1 (FigureS1C). Thus, the metal transport function was impaired in H_192_A in transgenic Arabidopsis.

A, Phenotype of Vector (GFP), MxIRT1 (MxIRT1-GFP), and three MxIRT1 His site-directed mutagenesis (HnA-GFP) transgenic and WT *Arabidopsis thaliana* after 30 days growth in soil. Scale bar, 5 cm.B, WB detecting the expression of Vector, MxIRT1 and MxIRT1 HRM point mutants using GFP antibody in transgenic *Arabidopsis thaliana*.C, Shoot length (cm) and chlorophyll content (SPAD index) in 30-day-old *Arabidopsis thaliana*. The Chlorophyll was measured in 30-day-old *Arabidopsis thaliana* leaves by SPAD-502 Chlorophyll Meter. Every independent measurement contains three replicates. Bars represent mean ± standard errors (n = 3). Different letters indicate significant differences (*P* < .05) as determined by ANOVA.D, Subcellular localization of Vector, MxIRT1, MxIRT1ΔHM and three MxIRT1 His site-directed mutants in rice protoplasts. GFP, green fluorescence protein indicates the location of fusion proteins; FM4-64, cell endomembrane system dye indicates of the cell membrane; Merge, image overlap showing both green fluorescence and red fluorescence; DIC, differential interference contrast. Fluorescence was observed after adding FM4-64 with 30 min. Scale bar, 10 µm.

Confocal results showed that MxIRT1 and H_184_A could target the PM in rice protoplasts (Figure 4d). Similar to MxIRT1∆HM, H_192_A and H_184-192_A disappeared within the cytoplasm. Thus, H192 among the loop is the vital site for determining MxIRT1 fusion with the PM in plants. These results indicate that H192 in HRM is required for the metal sensing function of MxIRT1 and fusion with the PM in transgenic plants.

### H192 in HRM determines the interaction of the loop of MxIRT1 with Fe^2+^

**Figure 5.**
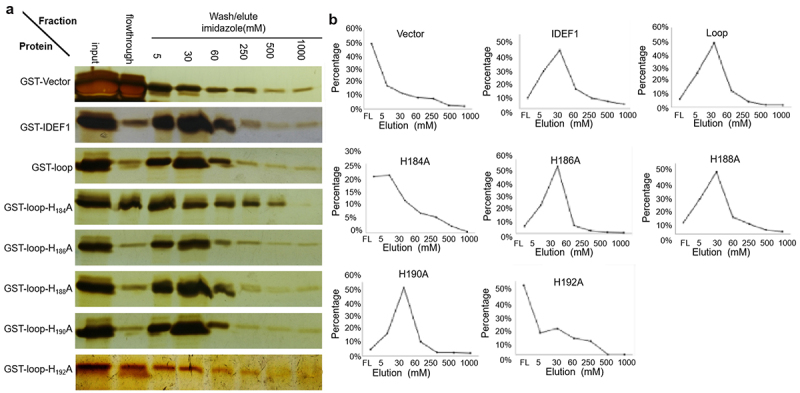
Iron-binding analysis of MxIRT1 HRM point mutants using immobilized metal ion affinity chromatography (IMAC).

To further analyze the function of H192, we performed metal-binding analysis using immobilized metal ion affinity chromatography (IMAC). When the columns were charged with Fe^2+^, the GST-vector fusion protein mostly passed through the column, indicating the low basal activity of GST binding to Fe^2+^ (Figure 5a). In contrast, the positive control GST-IDEF1 protein was trapped in the column and eluted only with high concentrations of imidazole, confirming that the GST-IDEF1 protein binds to Fe^2+^. The loop of MxIRT1 (domain between TMD3 and TMD4) showed a clear elution peak at high imidazole concentrations similar to the GST-IDEF1 protein, revealing a high binding capacity for these metals. Loop mutants H_184_A, H_186_A, H_188_A, and H_190_A also exhibited differing degrees of Fe^2+^ binding capacity, in accordance with the GST-loop. It is a remarkable fact that H_192_A showed broader and less steep peaks among the fractions, thus exhibiting lower binding than FL IDEF1. Most of the H_192_A passed through the column under 5 mM imidazole (Figure 5b), showing that H_192_A had the lowest Fe^2+^ binding capacity among the loop derivatives tested.

A, IMAC detecting the affinity of MxIRT1 and MxIRT1 HRM mutants with iron. The columns were charged with FeSO_4_ (Fe^2+^). The loop of MxIRT1 and its mutants fused to GST, GST as a negative control, or GST fused to IDEF1 as a positive control, were loaded. The columns were washed to remove the unbound proteins. Bound proteins were eluted with increasing concentrations of imidazole. Aliquots were subjected to sodium dodecyl sulfate-polyacrylamide gel electrophoresis and detected using fluorescent staining.B, Percentage of protein in each eluted aliquot.

### Y266 in the CRAC motif activates the metal transport ability of MxIRT1 under Fe deficient conditions

**Figure 6.**
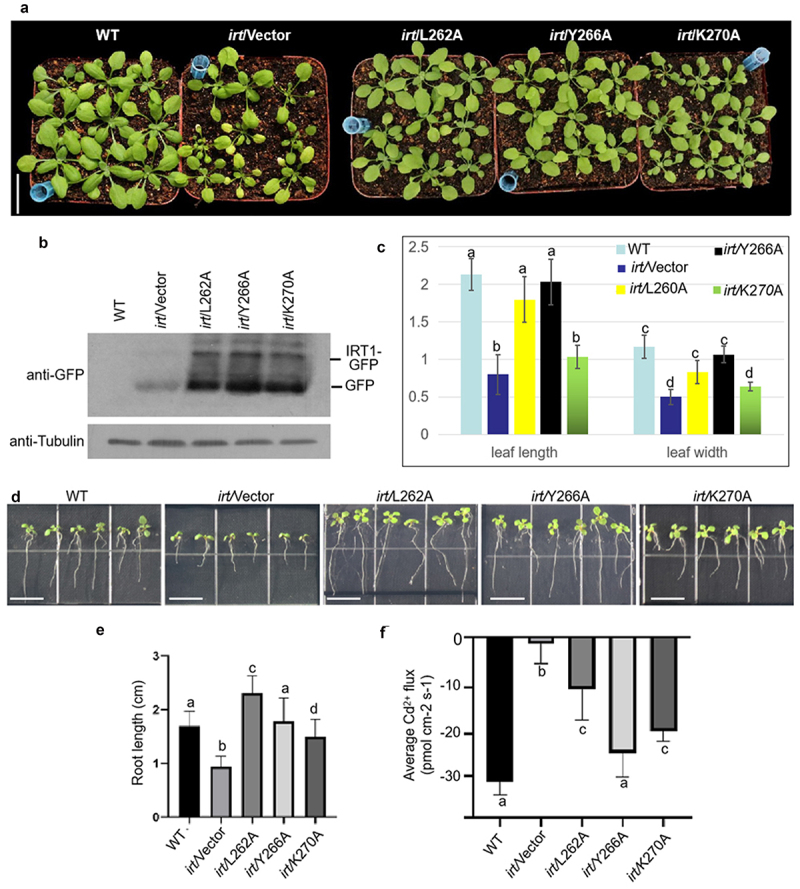
Phenotypic and functional analysis of Vector (GFP), MxIRT1 CRAC motif site-directed mutagenesis (L262A-GFP, Y266A-GFP, K270A-GFP) transgenic and WT *Arabidopsis thaliana* under iron sufficient condition.

Although we confirmed that the unique H192 in the loop of MxIRT1 sensed environmental iron through direct binding with iron and determined the fusion of MxIRT1 vesicles with the PM, whether MxIRT1 executed iron absorption immediately because of iron supply and iron binding remained unknown. In this study, we enhanced the mechanism regulating MxIRT1 function in the PM, based on our previous work. After MxIRT1 was distributed in the PM, we identified that the CRAC mutant Y266A could promote the movement of MxIRT1 from DRMs to non-DRMs, and this increased metal transport ability in yeast and rice protoplasts.^21^ To further identify the function of CRAC mutants in plants and their response to changes in iron concentration, we constructed the CRAC point mutants L262A, K270A, and Y266A in transgenic Arabidopsis. All CRAC transgenic Arabidopsis plants rescued the growth of *irt1* (Figure 6a). Western blotting was used to detect the protein expression of Vectors (GFP), L262A, K270A, and Y266A in Arabidopsis using GFP antibody (Figure 6b). In soil, these CRAC mutants induced some changes in leaf length and width in the aboveground part of the seedlings under iron-sufficient conditions, as compared to in WT, but no changes were observed with Y266A (Figure 6c). To observe the roots of transgenic Arabidopsis, we sowed the seeds in 1/2 MS. The phenotype of CRAC point mutants grown in 1/2 MS conditions were similar to those grown in soil (Figure 6d). Y266A grew at rates similar to WT under iron-sufficient conditions (Figure 6e). The average Cd^2+^ efflux of Y266A transgenic Arabidopsis was also the same as that of WT, and higher than that of L262A and K270A transgenic Arabidopsis under normal conditions, consistent with the previous results in yeast and rice protoplasts.^21^

A, The seedling phenotype of 30-day-old WT and CRAC site-directed mutagenesis transgenic *Arabidopsis thaliana* grown in soil.B, WB detecting the expression of Vector and CRAC motif point mutants using GFP antibody in transgenic *Arabidopsis thaliana*.C, Leaf length and leaf width in the 30-day-old *Arabidopsis thaliana* in soil. Every independent measurement contains 3 replicates. Bars represent mean ± standard errors (n = 3). Different letters indicate significant differences (*P* < .05) as determined using ANOVA.D, The root phenotype of 10-day-old Vector (GFP), CRAC point mutagenesis transgenic, and WT *Arabidopsis thaliana* in 1/2 MS medium. Transgenic seeds were sown and grown in 1/2 MS medium.E, Root length of Vector, MxIRT1 CRAC motif point mutagenesis transgenic and WT *Arabidopsis thaliana* in 1/2 MS medium. Every independent measurement contains three replicates. Bars represent mean ± standard errors (n = 3); Different letters indicate significant differences (*P* < .05) as determined using ANOVA.F, Comparison showing the average Cd^2+^ flux of MxIRT1 CRAC motif point mutagenesis transgenic *Arabidopsis thaliana* root under iron sufficient conditions. Every independent measurement contains >80 replicates. Bars represent mean ± SD. Different letters indicate significant differences (*P* < .05) as determined using ANOVA.

**Figure 7.**
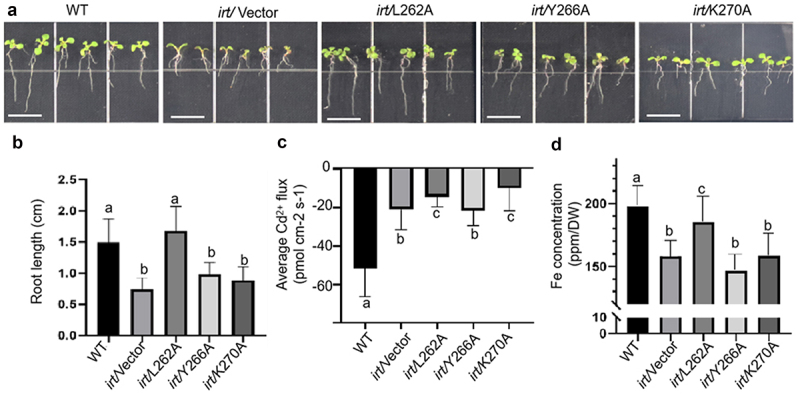
Phenotypic and functional analysis of Vector (GFP), MxIRT1 CRAC motif site-directed mutagenesis (L262A-GFP, Y266A-GFP, K270A-GFP) transgenic and WT *Arabidopsis thaliana* under iron deficient conditions.

To analyze the function of CRAC in response to iron changes, we compared the phenotype and Cd^2+^ flux of CRAC point mutants under different iron concentrations, including iron deficient (Figure 7a) and excess iron conditions (FigureS2A). Under iron-deficient conditions, L266A displayed the longest root length, while Y266A and K270A grew at rates similar to *irt1* (Figure 7b). From the Cd^2+^ flux test, we determined that CRAC mutants displayed low metal transport ability similar to *irt1* under iron-deficient conditions (Figure 7c). The iron concentration in CRAC mutants was also lower than that in WT (Figure 7d), which was consistent with previous results that the fusion of MxIRT1 vesicles with the PM requires iron stimulation.^18^ Thus, Y266 was sensitive to iron deficiency in the environment, as Y266A displayed the same root length (Figure 6d) and average Cd^2+^ flux (Figure 7c) as WT under iron-sufficient conditions, while exhibiting similar root length (Figure 7b), average Cd^2+^ flux (Figure 7c), and Fe concentration (Figure 7d) to mutant *irt1* under iron-deficient conditions.

A, The phenotype of 10-day-old Vector (GFP), CRAC point mutagenesis transgenic and WT *Arabidopsis thaliana* under iron deficient conditions. Transgenic seeds were sown in normal condition, and moved to iron deficient conditions (1/2 MS + 300 µM Ferrozine) after 3 days of germination.B, Root length of Vector, MxIRT1 CRAC motif point mutagenesis transgenic and WT *Arabidopsis thaliana* in iron deficient medium. Every independent measurement contains three replicates. Bars represent mean ± standard errors (n = 3). Different letters indicate significant differences (*P* < .05) as determined using ANOVA.C, Compare the average Cd^2+^ flux of MxIRT1 CRAC motif point mutagenesis transgenic *Arabidopsis thaliana* root under iron deficient conditions. Every independent measurement contains >80 replicates. Bars represent mean ± SD. Different letters indicate significant differences (*P* < .05) as determined using ANOVA.D, Fe concentration of Vector, MxIRT1 CRAC motif point mutagenesis transgenic, and WT *Arabidopsis thaliana* in iron deficient medium using ICP-MS. Every independent measurement contains three replicates. Bars represent mean ± standard errors (n = 3). Different letters indicate significant differences (*P* < .05) as determined using ANOVA.

Under excessive iron conditions (50 μM FeSO_4_), K270A displayed the shortest root length, which might be related to IRT1 ubiquitination, as its replacement causes iron toxic under excess iron conditions (Figure S2). Y266A displayed the lowest Cd^2+^ efflux under iron-rich conditions. As the endocytosis of MxIRT1 occurs in the non-DRM,^21^ the movement to non-DRM might enhance the endocytosis of MxIRT1 under excess iron conditions, which leads to a reduction in metal transport ability. These results indicate that Y266 is a critical site for sensing iron deficiency in the PM to regulate MxIRT1 function in plants.

## Discussion

The PM represents the border limiting the free flow of materials and acts as a checkpoint for the perception and integration of extracellular signals prior to signal transduction in living cells. Metals are taken up by several families of metal transporters in plants, and IRT1 represents the major entry route for Fe in plants while also mediating the unwanted acquisition of highly reactive Mn, Zn, Co, and Cd ions that tend to over-accumulate in plant tissues upon Fe deficiency.^2^ Therefore, research on the precise control of IRT1 localization and function has drawn much attention, and IRT1 has been established as one of the key model proteins for studying endomembrane trafficking in the last decade.^28^ Here, we identified that the unique H192 in HRM of MxIRT1 acted as an iron sensor by directly binding to iron and determining fusion with the PM, and Y266 activated metal transport in the PM under iron-deficient conditions. In our previous results, we found that most MxIRT1-vesicles were transmitted toward the PM and docked inside the cell, but without fusion in the absence of iron. However, by supplying iron to the environment, the fusion of MxIRT1-vesicles with the PM was promoted.^18^ Thus, the binding of iron may change the structure of the HRM, affecting its interaction with vesicles which promotes fusion with PM. Except for IRT1, how changes in extracellular iron affect the intracellular iron concentration may require further research. SNC1 encodes a component of the exocytosis machinery required for the fusion of Golgi-derived vesicles with the PM.^29^ SSO1 encodes a PM receptor protein involved in the fusion of secretory vesicles with the PM.^30^ In yeast mutants *Δsnc1* and *Δsso1*, MxIRT1 was not targeted to the PM and accumulated in secretory vesicles.^21^ Whether the binding of HRM with iron affects the interaction of MxIRT1-vesicle with SSO1 or SNC1 requires further research.

It is well known that histidine can effectively bind divalent ions, such as Fe^2+^, Zn^2+^, Ni^2+^, and Cu^2+.31–33^ Here, we found that the MxIRT1 derivative H192A lost the ability to bind to iron as detected by IMAC (Figure 5), which indicated that H192 could interact with iron directly, but the other mutants (H_184_A, H_186_A, H_188_A, H_190_A) could not achieve this interaction (Figure 5). However, mutants H_186_A, H_188/190_A could still affect the transport ability of MxIRT1,^20^ and the function of H_186_A and H_188/190_A might be related to metal influx after MxIRT1 fusion with the PM. The structure of the histidine of MxIRT1 and its binding to iron require further investigation to improve its affinity with iron. AtIRT1 has been regarded as a bifunctional transporter capable of directly binding and sensing non-iron metal availability using a histidine-rich motif in an intrinsically disordered cytosolic loop,^7^ but the IRT1 response to iron conditions remains unknown. The direct interaction of H192 with iron and the subsequent function in regulating fusion of MxIRT1 vesicles with the PM indicated that H192 or HRM was the iron sensor in MxIRT1. A previous study showed that MxIRT1 had the highest Fe and Zn uptake capacity, but the lowest Cd uptake capacity as compared to AtIRT1 and OsIRT1 in transgenic rice protoplasts.^21^ The unique existence of iron sensor H192 in MxIRT1 could explain the high efficiency of iron transport in M. *xiaojinensis*.

MxIRT1 is a member of the IRT1 family and displays the same function as other IRT1s, such as AtIRT1. Both have broad metal transport ability and play a key role in iron homeostasis in plants.^2,20^ There were also some non-negligible differences between MxIRT1 and AtIRT1. AtIRT1 is not subjected to iron-dependent degradation,^10^ whereas MxIRT1 proteins in transgenic yeast cells are subject to degradation through autophagy in the presence of excessive iron.^19^ AtIRT1 acts as a transceptor, directly sensing non-iron metal substrates to regulate its endocytosis and degradation;^7^ replacing histidines in the HRM has no effect on AtIRT1 metal transport.^34^ The direct interaction between AtIRT1 and Fe requires further research. However, the HRM is necessary for MxIRT1 iron transport,^20^ and can bind to Fe directly (Figure 5). These differences may be caused by the sequence of structural differences among various IRT1s.

After MxIRT1 is targeted to the PM, it is distributed in the DRM domain and might be dephosphorylated and moved to non-DRMs;^21^ we further found that Y266 in CRAC was necessary to activate the metal transport ability of MxIRT1 under iron-deficient conditions (Figure 7c). To analyze the mechanism by which CRAC regulates the interaction of MxIRT1 with cholesterol, which is the main component of DRMs, we predicted the structure of CRAC mutants using Discovery Studio software (Figure S3). L266 seemed to be the critical site for interaction with cholesterol, as it was closest to cholesterol. Substitution of L266 with Ala could increase the distance between the CRAC motif and cholesterol, while replacement of other CRAC motif sites did not change this distance, indicating that L266 was an important site for the association of MxIRT1 with cholesterol or lipid rafts. However, it is noteworthy that while L266 is responsible for the association with lipid rafts, it appears insensitive to iron changes. This indicates that L266A disassociates with lipid rafts depending on other iron-sensitive amino acids or that its activation requires signal transduction from the iron sensor. As the Y266 in CRAC lacks an iron sense, it may pass the signal of iron deficiency to L266. The CRAC motif ((L/V)-X_1–5_-(Y)-X_1–5_-(K/R)) could be considered a chemical fingerprint of cholesterol. Each of the three amino acid residues that define the CRAC motif has a specific function in cholesterol recognition. The N-terminal branched residue (valine or leucine) binds to the iso-octyl chain of cholesterol through van der Waals interactions. In contrast, the C-terminal polar residue (lysine or arginine) faces the OH group of cholesterol, allowing the formation of a hydrogen bond.^35^ Based on our predicted structure and optimized phenotype of the whole iron condition of CRAC mutants, L266A is also involved in DRM interaction, but does not participate in the iron response. Y266A transgenic Arabidopsis displayed a severe chlorosis phenotype similar to *irt1*, indicating the critical role of Y266 in the CRAC motif in response to iron deficiency. As Y266 has been predicted to be a phosphorylation site and displacement of Y266 promotes disassociation with DRMs and activates metal transport,^21^ the role of Y266 in sensing iron deficiency illustrates that MxIRT1 may sense iron deficiency in DRMs, dephosphorylates and move to non-DRMs to active metal transport. The K270A mutant presented a sluggish phenotype under excess iron condition, which implied that K270 might negatively regulate CRAC metal transport. Thus, we speculate that the dephosphorylation of Y266 and ubiquitination of K270 are important for regulating the disassociation of L262A with cholesterol and movement of MxIRT1 from DRMs to non-DRMs; however, this requires further research.

Using co-immunopurification (co-IP) of IRT1 and identification of IRT1-interacting proteins by mass spectrometry, Martin-Barranco et al. 2020 shed light on the existence of a dedicated protein complex composed of IRT1, FRO2, and AHA2, which likely optimizes iron uptake in the PM of root epidermal cells; IRT1 is then selectively removed from the complex in response to non-iron metal excess, in a process involving its phosphorylation.^36^ Although detergent-resistant membranes (DRMs, a biochemical counterpart of PM microdomains) are known platforms for signal transduction in the PM and act as a component of a mechanism for protein sorting and trafficking in plants to promote regulation of iron homeostasis, the complex composed of IRT1, FRO2, and AHA2 may be formed in the DRMs as IRT1 targeting to the PM is regulated by DRMs. After MxIRT1 was targeted to the PM, it was distributed in the DRM domain and might be dephosphorylated and move to non-DRMs,^21^ along with complex disassembly. After AtIRT1 targets to the PM, it is ubiquitinated at K154/179 and endocytosis into the intracellular compartment is regulated by non-iron divalent metals (Zn, Mn, Co).^9^ Further investigation is needed to clarify this model and the relationship between ubiquitination and phosphorylation. Hence, H192, a site specific to MxIRT1, in the HRM acts as an iron sensor to determine the delivery of MxIRT1 vesicles to the PM after binding with iron directly, after which Y266 in CRAC senses iron deficiency to active metal transport.

## Supplementary Material

Supplemental Material
