# Supplementary material for: HRM and CRAC in MxIRT1 act as iron sensors to determine MxIRT1 vesicle-PM fusion and metal transport
Source: Plant Signal Behav. 2021 Nov 23;17(1):2005881. doi: 10.1080/15592324.2021.2005881 (PMC8928839; doi:10.1080/15592324.2021.2005881)
Supplement: Supplemental Material [file KPSB_A_2005881_SM0587.doc]

**Supplemental Tables**

**Table S1. Primers name and sequence**

**Supplemental Figures**

**Figure S1. Phenotypic analysis of HRM mutagenesis transgenic and WT *Arabidopsis thaliana* after 7 days growth in 1/2 MS.**

**A,** Phenotype of Vector, MxIRT1, and three MxIRT1 His site-directed mutagenesis transgenic and WT *Arabidopsis thaliana* after 7 days growth in 1/2 MS. Scale bar, 1cm.

**B,** Root length (cm) in 7-day-old *Arabidopsis thaliana*. Every independent measurement contains 3 replicates. Bars represent mean ± standard errors (n = 3). Different letters indicate significant differences (P<0.05) as determined using ANOVA.

**C,** Leaf area in 7-day-old *Arabidopsis thaliana*. Every independent measurement contains 3 replicates. Bars represent mean ± standard errors (n = 3). Different letters indicate significant differences (P<0.05) as determined using ANOVA.

**Figure S2. Phenotypic and functional analysis of Vector (GFP), MxIRT1 CRAC motif site-directed mutagenesis (L262A-GFP, Y266A-GFP, K270A-GFP) transgenic and WT *Arabidopsis thaliana* under excess iron conditions (1/2MS + 50 µM FeSO_4_).**

**A,** The phenotype of 10-day-old Vector (GFP), CRAC point mutagenesis transgenic and WT *Arabidopsis thaliana* under excess iron conditions. Transgenic seeds were sown in normal condition, and moved to excess iron condition after 3 days of germination. Scale bar, 1cm.

**B,** Root length of Vector, MxIRT1 CRAC motif point mutagenesis transgenic and WT *Arabidopsis thaliana* under excess iron conditions. Every independent measurement contains 3 replicates. Bars represent mean ± standard errors (n = 3). Different letters indicate significant differences (P<0.05) as determined using ANOVA.

**C,** Compare the average Cd^2+^ flux of MxIRT1 CRAC motif point mutagenesis transgenic Arabidopsis thaliana root under excess iron conditions. Every independent measurement contains >80 replicates. Bars represent mean ± SD. Different letters indicate significant differences (P<0.05) as determined using ANOVA.

**Figure S3. Predict the structure of MxIRT1 and CRAC point mutants, and their interaction with cholesterol.** The structures are predicted and analyzed by Discovery Studio. The helix represents amino acids of MxIRT1. The blue grid represents cholesterol. The red grid represents CRAC motif among the MxIRT1.

***Table S1. Primers name and sequence***

| **Primer name** | **Primer sequence** |
| --- | --- |
| MxIRT1-F | 5’-CTTCCCTCTTGTCACTCGTT-3’ |
| MxIRT1-R | 5’-TATCATTGTCCATTCAGTTGTTAT-3’ |
| H (Up-down) | 5’-CCCAGCACCCACCACAGCCATCTCT-3’ |
| H (Down-up) | 5’-GTGGTGGGTGCTGGGGTTGTTGACAAGGGAGAAAACGGAGACTCG-3’ |
| H_184_A-F | 5’-GTGGGTGCTGGGGCTGGCCATTTTCAT-3’ |
| H_184_A-R | 5’-ATGAAAATGGCCAGCCCCAGCACCCAC-3’ |
| H_192_A-F | 5’-CATGCACACAACGCTGTTGTTGAC-3’ |
| H_192_A-R | 5’-GTCAACAACAGCGTTGTGTGCATG-3’ |
| H_184/192_A-F | 5’-GCTGCAGCCAACGCTGTTGTTGACAAG-3’ |
| H_184/192_A-R | 5’-CTTGTCAACAACAGCGTTGGCTGCAGC-3’ |
| H_184-192_A-F | 5’-CTGGGCATGGCGCTTTTGCTG-3’ |
| H_184-192_A-R | 5’-CAGCAAAAGCGCCATGCCCAG-3’ |
| L262A-F | 5’-GGCGGTTGCATTGCACAGGCCGAGTAC-3’ |
| L262A-R | 5’-GTACTCGGCCTGTGCAATGCAACCGCC-3’ |
| Y266A-F | 5’-CTACAGGCCGAGGCCAAGTTCATGAAG-3’ |
| Y266A-R | 5’-CTTCATGAACTTGGCCTCGGCCTGTAG-3’ |
| K270A-F | 5’-TACAAGTTCATGGCGAAGGCCATAATG-3’ |
| K270A-R | *5’-CATTATGGCCTTCGCCATGAACTTGTA-3’* |

**Figure S1. Phenotypic analysis of HRM mutagenesis transgenic and WT *Arabidopsis thaliana* after 7 days growth in 1/2 MS.**


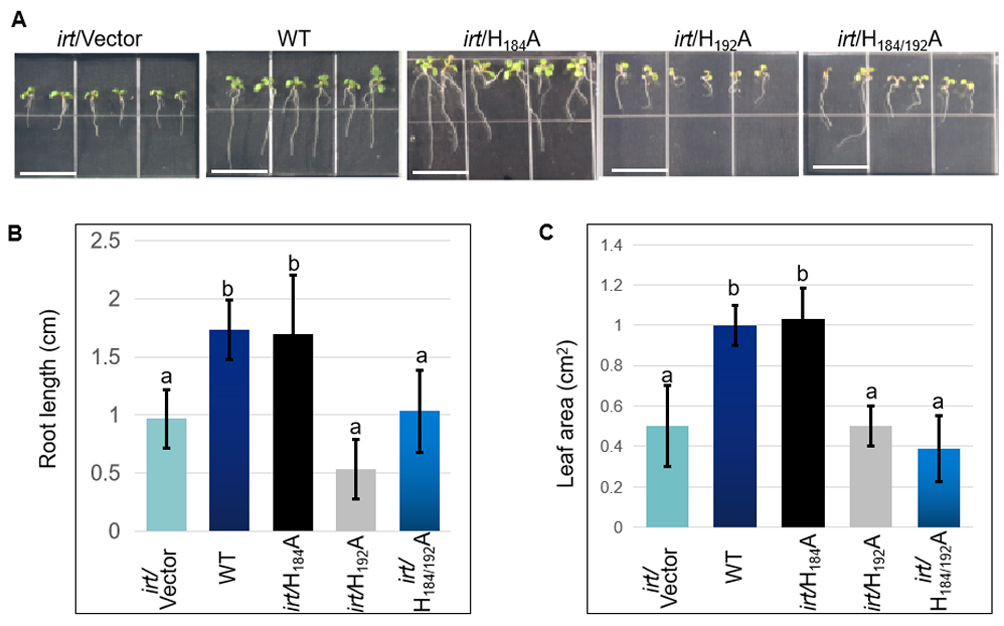


**Figure S2. Phenotypic and functional analysis of Vector (GFP), MxIRT1 CRAC motif site-directed mutagenesis (L262A-GFP, Y266A-GFP, K270A-GFP) transgenic and WT *Arabidopsis thaliana* under excess iron conditions (1/2 MS + 50 µM FeSO_4_).**


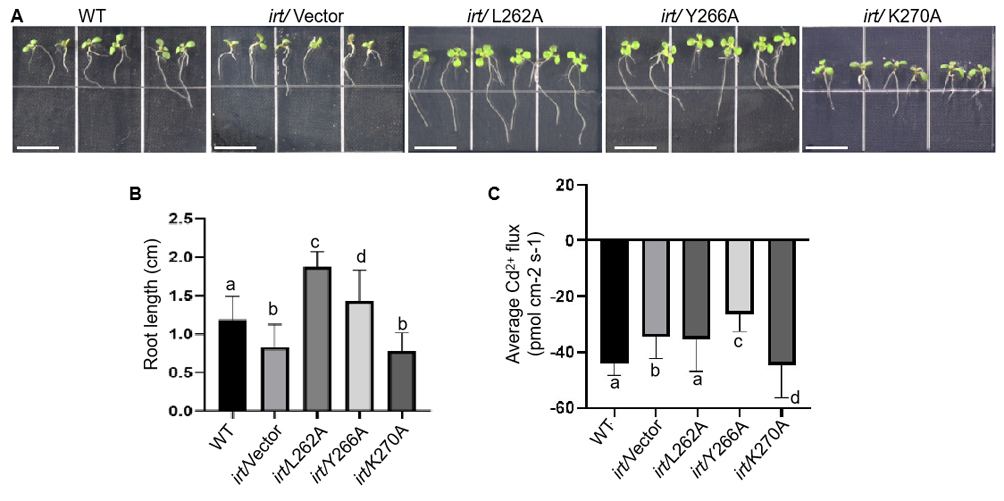


**Figure S3. Predict the structure of MxIRT1 and CRAC point mutants, and their interaction with cholesterol.** The structures are predicted and analyzed by Discovery Studio. The helix represents amino acids of MxIRT1. The blue grid represents cholesterol. The red grid represents CRAC motif among the MxIRT1.

**
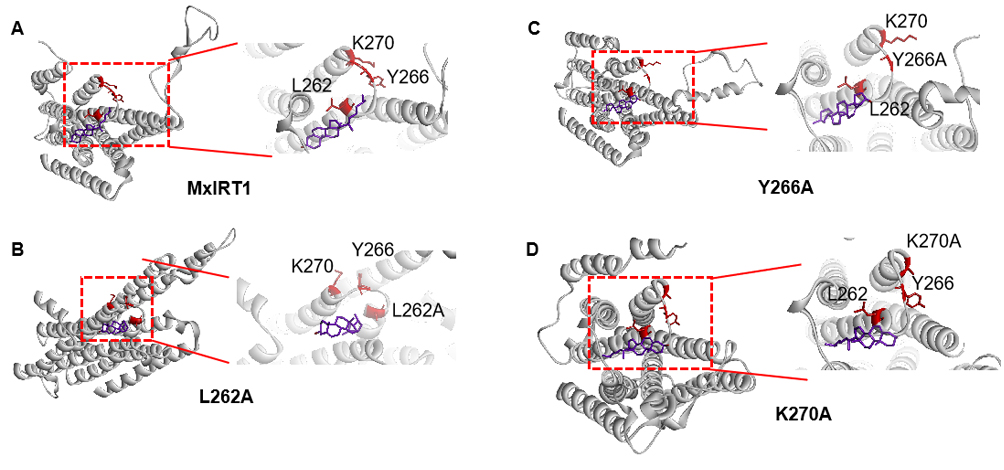
**
